# Whole-Transcriptome Analysis of Non-Coding RNA Alteration in Porcine Alveolar Macrophage Exposed to Aflatoxin B1

**DOI:** 10.3390/toxins14060373

**Published:** 2022-05-27

**Authors:** Huhe Chao, Haohai Ma, Jiadong Sun, Shuai Yuan, Peiyu Dong, Aihong Zhao, Lan Li, Wei Shen, Xifeng Zhang

**Affiliations:** 1 College of Veterinary Medicine, Qingdao Agricultural University, Qingdao 266109, China; chaohuhe@126.com (H.C.); 20202113033@stu.qau.edu.cn (H.M.); 20212213004@stu.qau.edu.cn (P.D.); 2 Central Laboratory, Beijing Obstetrics and Gynecology Hospital, Capital Medical University, Beijing 100023, China; 3 Key Laboratory of Animal Reproduction and Biotechnology in Universities of Shandong, College of Life Sciences, Qingdao Agricultural University, Qingdao 266109, China; sun-jiadong@163.com (J.S.); lli@qau.edu.cn (L.L.); wshen@qau.edu.cn (W.S.); 4 School of Medicine, Henan Polytechnic University, Jiaozuo 454000, China; wsys727@sina.com; 5 Qingdao Academy of Agricultural Science, Qingdao 266100, China; zhaoaihong0@163.com

**Keywords:** aflatoxin B1, porcine, porcine alveolar macrophages, apoptosis, cell cycle

## Abstract

Aflatoxin B1 (AFB1) is a type of mycotoxin produced by the fungi Aspergillus flavus and Aspergillus parasiticus and is commonly found in cereals, oils and foodstuffs. In order to understand the toxic effects of AFB1 exposure on Porcine alveolar macrophages (3D4/2 cell), the 3D4/2 cells were exposed to 40 μg/mL AFB1 for 24 h in vitro, and several methods were used for analysis. Edu and TUNEL analysis showed that the proliferation of 3D4/2 cells was significantly inhibited and the apoptosis of 3D4/2 cells was significantly induced after AFB1 exposure compared with that of the control group. Whole-transcriptome analysis was performed to reveal the non-coding RNA alteration in 3D4/2 cells after AFB1 exposure. It was found that the expression of cell-cycle-related and apoptosis-related genes was altered after AFB1 exposure, and lncRNAs and miRNAs were also significantly different among the experimental groups. In particular, AFB1 exposure affected the expression of lncRNAs associated with cellular senescence signaling pathways, such as MSTRG.24315 and MSTRG.80767, as well as related genes, Cxcl8 and Gadd45g. In addition, AFB1 exposure affected the expression of miRNAs associated with immune-related genes, such as miR-181a, miR-331-3p and miR-342, as well as immune-related genes Nfkb1 and Rras2. Moreover, the regulation networks between mRNA-miRNAs and mRNA-lncRNAs were confirmed by the results of RT-qPCR and immunofluorescence. In conclusion, our results here demonstrate that AFB1 exposure impaired proliferation of 3D4/2 cells via the non-coding RNA-mediated pathway.

## 1. Introduction

Mycotoxin is the toxic metabolite of mold, which is limited to some strains of a few toxigenic molds. Different molds can produce the same mycotoxin, while one strain can produce several mycotoxins [1]. At present, about 200 kinds of mycotoxins have been found, and a few of them can cause poisoning in animals and humans under natural conditions [2]. The most important mycotoxins are aflatoxin B1, ochratoxin A, zearalenone, fumin and deoxynivalenol. Mycotoxins can pollute all types of food and feed and can threaten human and animal health through food chain accumulation, producing hepatotoxicity, nephrotoxicity, neurotoxicity, hematopoietic tissue toxicity, etc. [3,4,5]. Some mycotoxins are mutagenic and carcinogenic [6,7].

Aflatoxin B1 is the most toxic mycotoxin; it was listed as a Group 1 carcinogen by the International Agency for Research on Cancer (IARC) in 1996 [8]. AFB1 mainly targets the liver of humans and animals, where it is metabolized by cytochrome 450 into carcinogenic AFB1-8,9-exo-epoxide (AFBO). AFBO combines with phase II enzymes such as glutathione-S-transferase (GST) to form afb1-thiol acid (AFB1-NAC), which is excreted with urine. AFBO can also combine with DNA to form AFB1-N7-Gua, causing DNA mutation [9,10]. AFB1 reduces steroid production by competitively binding StAR protein of rats, affects the secretion of estradiol-17β and progesterone in animal serum, inhibits the growth of oocytes and leads to the decrease of ovarian size and weight [11,12]. In male mice, AFB1 exposure is related to histological changes of testis, reduction of sperm number and differences in sperm motility and litter size [13,14]. In primary broiler hepatocytes, AFB1 results in an increase in mitochondrial ROS production, a decrease in mitochondrial membrane potential and an induction of apoptosis. This is related to the upregulation of *Nrf2* gene expression and downregulation of NAD(P)H: quinine oxidoreductase 1, SOD and HO-1 [15]. AFB1, as a potential endocrine disruptor, can affect the expression of aromatase enzymes (P450s or CYPs enzymes) [16].

Epigenetic modification includes DNA methylation, ncRNA (miRNA, lncRNA and circular RNA) and post-translational modification (PTM) (glycosylation, methylation, acetylation, phosphorylation and ubiquitination) [17,18]. ncRNA participates in various biological processes, and abnormal expression of ncRNA always destroys the balance in vivo and leads to diseases [19,20]. At present, most studies involving ncRNAs focus on miRNA, circRNA and lncRNA. LncRNA is involved in X chromosome silencing, genome imprinting, chromatin modification, transcription activation, transcription interference, nuclear transport and other important regulatory processes, such as apoptosis. LncRNA is often used to study toxicological mechanisms [21,22].

This study was designed to determine the mechanism of lncRNA and microRNA targeting regulatory genes in Porcine alveolar macrophage cells in response to the toxic effects of AFB1. We generated differential expression profiles of lncRNA and miRNA in Porcine alveolar macrophage cells with and without AFB1 exposure. The findings of this research provide the molecular mechanisms involved in the development of AFB1-induced hepatotoxicity and enrich the valuable resources for lncRNA and miRNA in toxicological research.

## 2. Results

### 2.1. AFB1 Inhibited Cell Proliferation and Induced Cell Apoptosis

**Figure 1:**
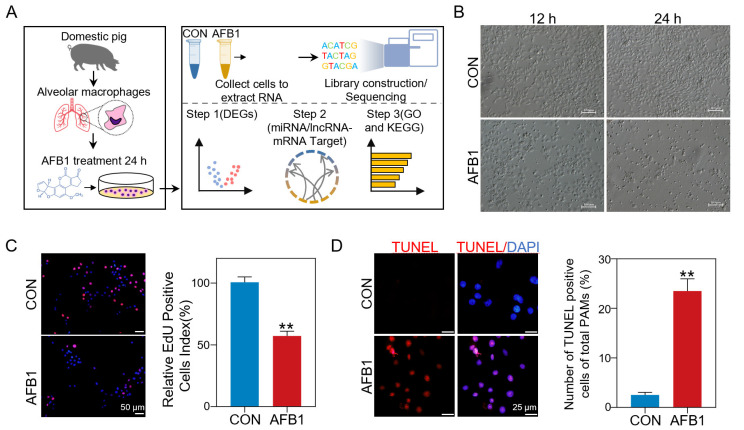
Sequence and data preprocessing of porcine 3D4/2 cell. (**A**) The Schematic diagram of sample treatment and RNA sequencing procedure. (**B**) The morphological changes of porcine 3D4/2 cells exposed to AFB1 for 12 and 24 h in vitro. Scale bar, 100 mm. (**C**) Representative immunofluorescent images of EdU positive cells (red) and the cell nuclei (blue) in the control and AFB1-exposed cells after 24 h (**left**), and the percentages of EdU positive cells (**right**). ** *p* < 0.01. (**D**) Percentages of TUNEL-positive cells treated for 24 h in different groups (**left**) and number of TUNEL-positive cells of the total cells (%, (**right**)). ** *p* < 0.01. All experiments were repeated 3 times.

EdU assay is a commonly used method for detecting cell proliferation. In order to deeply understand the molecular mechanism of porcine 3D4/2 cell cytotoxicity induced by AFB1 exposure, porcine 3D4/2 cells were treated with 40 μm AFB1 in vitro for 24 h. The whole experimental design is shown in Figure 1A. Compared with the untreated group, cells treated with 40 μm AFB1 had significant morphological differences (Figure 1B). The proliferation ability of porcine 3D4/2 cells was checked with an EdU kit, and the results showed that the number of EdU-positive cells in the AFB1 treatment groups decreased significantly compared with the control group (Figure 1C). After AFB1 treatment, the number of TUNEL-positive cells was significantly increased (23.5%) compared with the control group (2.2%) (Figure 1D).

### 2.2. AFB1 Exposure Affected lncRNA and mRNA Expression of Porcine 3D4/2 Cells

**Figure 2:**
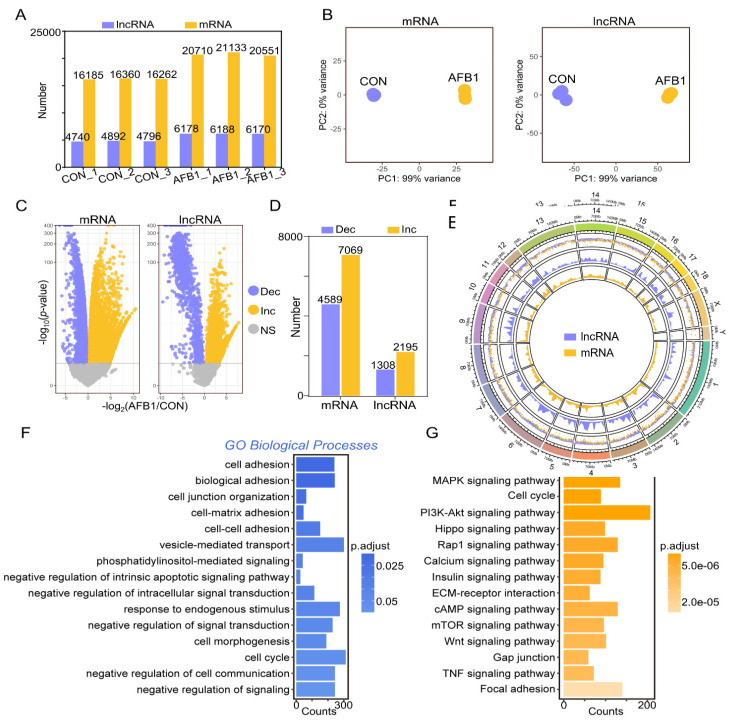
Divergent expression patterns of lncRNA and mRNA of porcine 3D4/2 cells. (**A**) The number of lncRNA and mRNA transcripts in control and AFB1-treated groups. (**B**) Principal component analysis (PCA) based on lncRNA and mRNA. (**C**) The volcano plots of differentially expressed genes (DEGs) and lncRNAs (DELs) of porcine 3D4/2 cells. (**D**) The number of upregulated and downregulated DEGs and DELs of control vs. AFB1-treated group. (**E**) The chord diagram showing the distribution and expression of differentially expressed lncRNAs and mRNAs in the chromosome. (**F**) GO and (**G**) KEGG enrichment of DEGs.

Ribonucleic acid sequencing (RNA-seq) was utilized to explore the effect of AFB1 on the expression of lncRNAs and mRNAs in porcine 3D4/2 cells. Figure 2A shows the number of lncRNA and mRNA transcripts in the control and AFB1-treated groups. Based on the principal component analysis (PCA), the various lncRNA and mRNA datasets with the same treated methods were highly similar, respectively (Figure 2B). According to the volcano plots of differentially expressed genes (DEmRNAs) and lncRNAs (DElncRNAs) of porcine 3D4/2 cells (Figure 2C), there were 4589 and 1308 downregulated mRNA and lncRNA in the control versus the AFB1-treated group, respectively, and the upregulated mRNAs and lncRNAs were 7069 and 2195, respectively (Figure 2D). The distribution and expression of each lncRNA and mRNA on each chromosome are displayed by the chord diagram (Figure 2E). Gene Ontology (GO) and Kyoto Encyclopedia of Genes and Genomes (KEGG) enrichment analyses were performed based on DEmRNAs. The enriched GO terms included cell adhesion, biological adhesion, vesicle mediated transport, response to endogenous stimulus, negative regulation of signal transduction, and others (Figure 2F). KEGG analysis was used to examine pathway enrichment (Figure 2G). The top 15 enriched KEGG pathways involved the MAPK signaling pathway, P13K-Akt signaling pathway, Hippo signaling pathway, Camp signaling pathway, mTOR signaling pathway, TNF signaling pathway and Focal adhesion (Figure 2G).

### 2.3. Co-Expression Analysis of DELs and DEGs in Porcine 3D4/2 Cells

**Figure 3:**
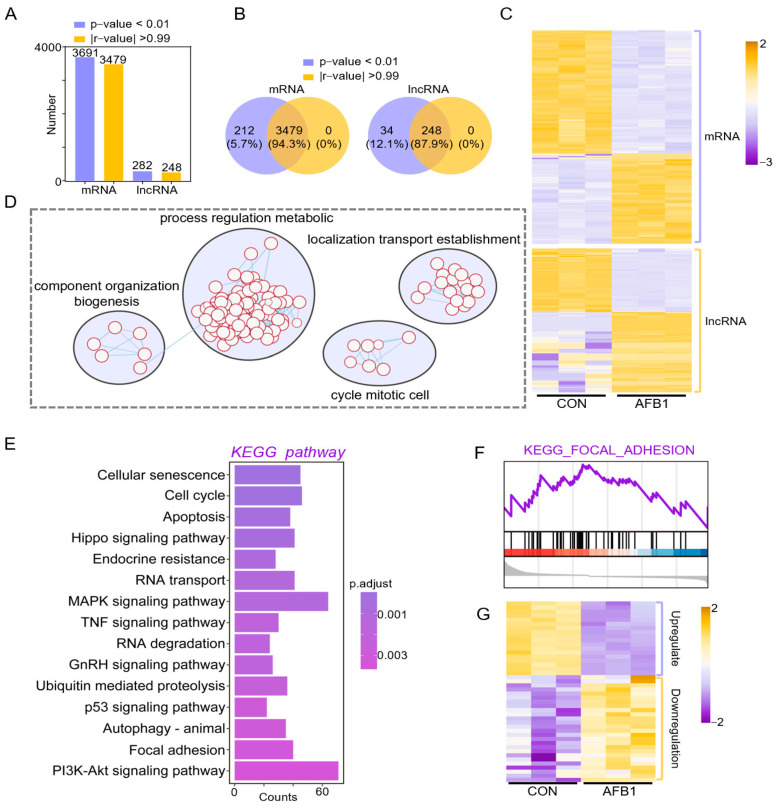
Co-expression analysis of DELs and DEGs in porcine 3D4/2 cells. (**A**) Identification of pairwise lncRNA-mRNA in co-expression analysis; the histogram showing the number of mRNA and lncRNA with *p*-value < 0.01 and |r-value| > 0.99. (**B**) The Venn diagram showing the number of mRNA and lncRNA with *p*-value < 0.01 and |r-value| > 0.99. (**C**) The heatmap showing the expression of mRNA and lncRNA. (**D**) Functional enrichment analysis of co-expressed genes. (**E**) KEGG enrichment results of co-expressed genes. (**F**) GSEA enrichment results of co-expressed genes. (**G**) The heatmap showing gene expression in the cellular senescence signaling pathway.

To accurately identify the regulatory mechanisms of lncRNAs and mRNAs, we performed co-expression analysis based on Differentially expressed genes (DEGs) and Differentially expressed lncRNAs(DELs). After filtering according to *p*-value (*p* < 0.01) and Pearson correlation coefficient, 3479 mRNAs and 248 lncRNAs were obtained (Figure 3A,B). The heatmap was plotted according to the expression of mRNA and lncRNA (Figure 3C), which were all related to component organization biogenesis, process regulation metabolic, cycle mitotic cell and localization transport establishment (Figure 3D). KEGG pathway analysis was performed to elucidate the function of co-expressed genes (Figure 3E). We obtained similar enrichment results as above, including cellular senescence, cell cycle, mitogen-activated protein kinase (MAPK) signaling pathway, Tumor necrosis factor (TNF) signaling pathway, p53 signaling pathway and phosphatidylinositol 3 kinase-protein kinase B (PI3K-Akt) signaling pathway, which were all related to apoptosis, indicating that AFB1 exposure affected mRNA and lncRNA expression of 3D4/2 cells and led to cell apoptosis (Figure 3E). For trend analysis of RNA data sets, we also performed GSEA analysis. The results showed that the gene expression related to the focal adhesion pathway was upregulated (Figure 3F). The heatmap shows the gene expression in cellular senescence signaling pathway (Figure 3G).

### 2.4. Cis-Regulation of mRNA and lncRNAs with Target Genes

**Figure 4:**
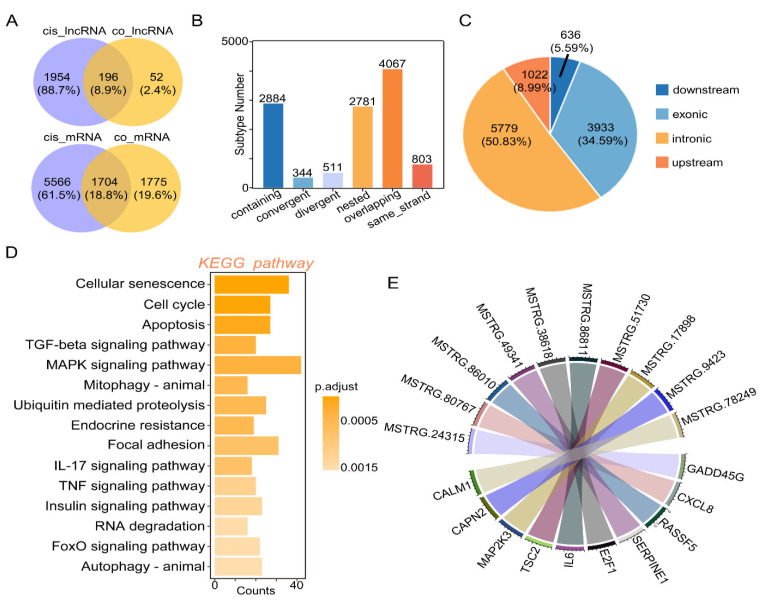
Cis-regulation of lncRNAs with target genes. (**A**) The Venn plot of unique lncRNAs or mRNAs in cis-regulation with co-expressed lncRNAs or mRNA. (**B**) Subtype statistics of key cis-regulatory mRNA. According to the illustration, “Genic” includes the subtypes of “overlapping”, “containing”, and “nested”; “Intergenic” consists of “same strand”, “convergent”, and “divergent” subtypes. (**C**) Genome location statistics of key cis-regulatory mRNA. (**D**) KEGG enrichment analysis of target genes regulated by lncRNA homeopathy. (**E**) The chord graph showing the targeting relationship between key genes in the cellular senescence signaling pathway and lncRNA.

**Figure 5:**
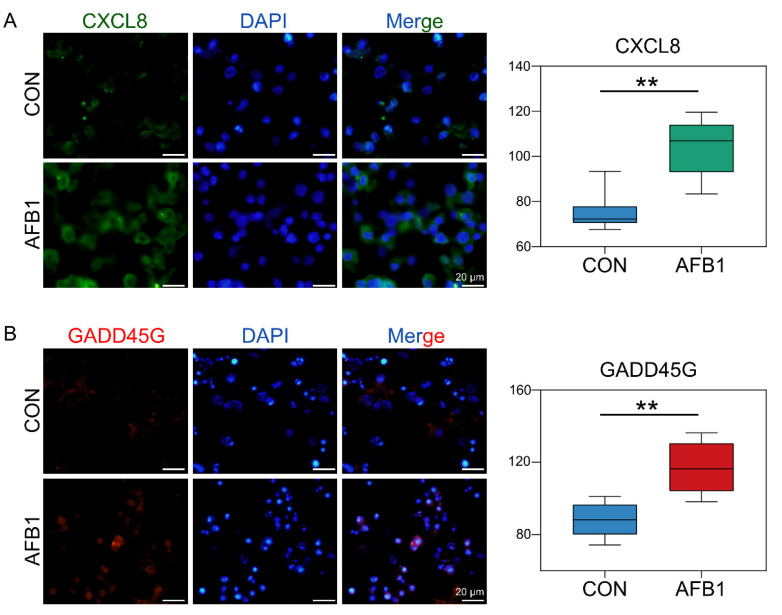
Cell immunofluorescence assay of the expression of CXCL8 and GADD45G proteins. (**A**) The fluorescence intensity and positive percentages of CXCL8. Nuclear staining (blue) and CXCL8-positive cells (green). (**B**) The fluorescence intensity and positive percentages of GADD45G. Nuclear staining (blue) and GADD45G-positive cells (red). ** Indicates extremely significant differences (*p* < 0.01). All experiments were repeated 3 times.

Based on the Venn plots of unique lncRNAs or mRNA in cis-regulation with co-expressed lncRNAs or mRNA, 196 key lncRNAs and 1704 key mRNAs were found (Figure 4A). Subtype statistics of key cis-regulatory mRNA were assayed. According to the illustration, “Genic” includes the subtypes of “overlapping”, “containing”, and “nested”; “Intergenic” consists of “same strand”, “convergent”, and “divergent” subtypes (Figure 4B). Genome location statistics showed that the key cis-regulatory mRNAs were located upstream (8.99%), intronic (50.83%), exonic (34.59%) and downstream (5.59%), respectively (Figure 4C). To understand the function of DElncRNAs target genes, we explored the function of these target genes using KEGG analysis (Figure 4D). KEGG analysis showed that there are 15 significantly enriched signal pathways with DElncRNAs, including cellular senescence, cell cycle, MAPK signaling pathway, IL-17 signaling pathway, TNF signaling pathway, autophagy, etc. (Figure 4D). Next, based on co-expression and co-localization analysis, we found that DElncRNAs regulated key genes in cellular senescence signaling pathways, and we display them through chord diagrams (Figure 4E). The relative expression of these genes was determined by Immunofluorescence Staining (Figure 5). Compared to the control group, the expression levels of CXCL8 and GADD45G were significantly upregulated (Figure 5), which was consistent with the whole transcriptome sequence results.

### 2.5. AFB1 Exposure Altered miRNA Expression

**Figure 6:**
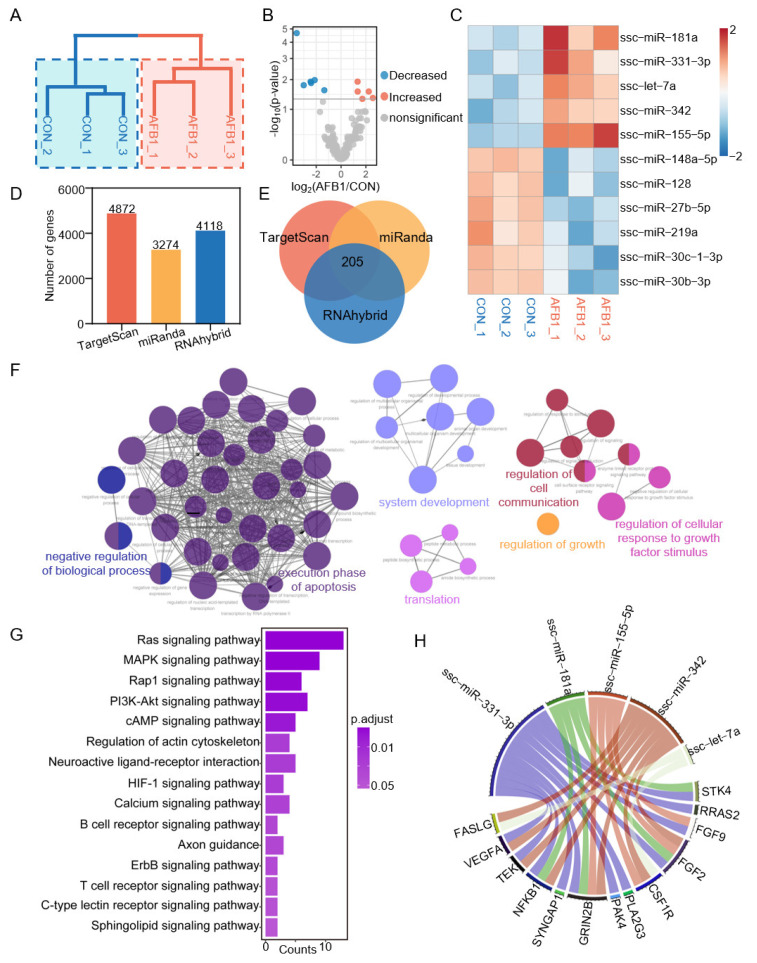
AFB1 exposure alters the miRNA expression levels of porcine 3D4/2 cells. (**A**) The cluster dendrogram between AFB1-treated groups and the control groups based on miRNA. (**B**) The volcano diagram showing the distribution of DEmiRNAs between the AFB1-treated group and the control group. (**C**) The heatmap demonstrating the expression level of DEmiRNAs in six samples. (**D**) The histogram showing the number of miRNAs and the number of target genes. (**E**) The Venn diagram showing the number of target genes shared by DEmiRNAs in TargetScan, miRanda and RNAhybrid. (**F**) GO enrichment analysis of DEmiRNA target genes. (**G**) KEGG enrichment analysis of DEmiRNA target genes. (**H**) The circos diagram indicating the targeting relationship of the miRNA-mRNA network in porcine 3D4/2 cells.

To investigate the impact of AFB1 on miRNA in porcine 3D4/2 cells, we performed differential expression analysis of miRNA between three control groups and three AFB1-treated groups (Figure 6A). The volcano plot was used to show the distribution of differentially expressed miRNAs (Figure 6B). Compared with the control group, a total of 5 DEmiRNAs were upregulated and 6 DEmiRNAs were downregulated in the AFB1-treated group. The change of DEmiRNA expression in the different groups is shown in the heatmap (Figure 6C). Moreover, TargetScan, miRanda and RNAhybrid software (http://www.targetscan.org/, http://www.microrna.org/microrna/home.do, and http://bibiserv.techfak.uni-bielefeld.de/rnahybrid/, accessed on 25 April 2022) was used for predicting the DEmiRNA-related genes (Figure 6D,E), and finally 205 genes were predicted (Figure 6E). The genes were found to be enriched in GO terms associated with the negative regulation of biological processes, execution phase of apoptosis, system development, translation, regulation of cell communication, regulation of growth and regulation of cellular response to growth factor stimulus (Figure 6F). The KEGG analysis showed that the target genes were enriched in the Ras signaling pathway, MAPK cell signaling pathway, Rap1 signaling pathway, P13k-Akt signaling pathway, cAMP signaling pathway, calcium signaling pathway, etc. (Figure 6G). We show the targeting relationship between key genes and miRNAs in the Ras signaling pathway (Figure 6H).

**Figure 7:**
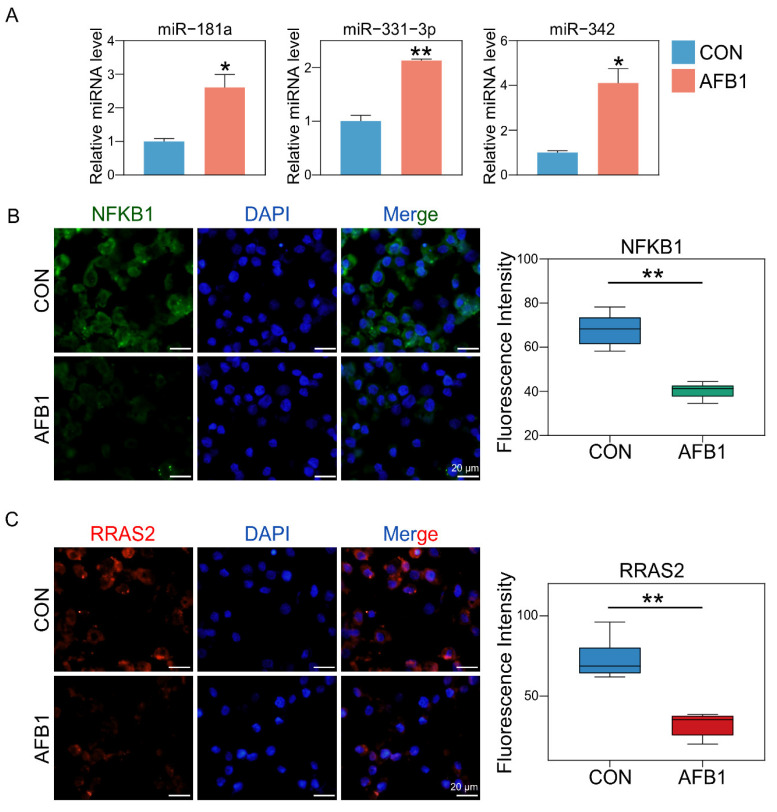
Validation of miRNA-seq data availability with RT-qPCR and examination of the expression of NFKB1 and RRAS2 proteins with cell immunofluorescence assay. (**A**) Expression of miR-181a, miR-331-3p and miR-342 in 3D4/2 cells after 24 h AFB1 exposure. * Indicates extremely significant differences (*p* < 0.05), ** Indicates extremely significant differences (*p* < 0.01). All experiments were repeated 3 times. (**B**) The fluorescence intensity and positive percentages of NFKB1. Nuclear staining (blue) and NFKB1 positive cells (green). (**C**) The fluorescence intensity and positive percentages of RRAS2. Nuclear staining (blue) and NFKB1 positive cells (red). ** Indicates extremely significant differences (*p* < 0.01). All experiments were repeated 3 times.

We selected genes shared among the DEmRNA and DEmiRNA target genes (Figure S2A). We use the heatmap to show the expression of key genes in different groups (Figure S2B). Subsequently, we performed KEGG enrichment analysis on the 74 target genes (Figure S2C). The results indicated that the Ras signaling pathway was mainly regulated by miRNAs. Using genetic interactions and co-expression networks, we found that NFKB1 and RRAS2 play key roles in the Ras signaling pathway (Figure S2D). The targeting relationships between miRNAs and key genes are shown in Figure S2E. The relative expression of DEmiRNAs was determined by RT-qPCR. Compared with the control group, the expression levels of miR-181a, miR-331-3p and miR-342 in the AFB1 treatment group were significantly upregulated (Figure 7A). To verify the expression of the previous related genes *Nfkb1* and *Rras2* after AFB1 exposure, we further analyzed their expression using immunofluorescence. The fluorescent intensity of NFKB1 and RRAS2 genes was significantly decreased in the AFB1-treated group compared with that of the control group (Figure 7B,C). These results were consistent with the data of the whole-transcriptome sequence.

## 3. Discussion

Aflatoxin can induce mutation, inhibit immunity and cause cancer. The liver tissue is the main target organ of aflatoxin, which can lead to liver cancer and even death in severe cases [23,24]. Acute poisoning of animals can lead to serious damage to blood vessels and the central nervous system, and animals may die within several hours to several days after poisoning. Chronic poisoning is characterized by poor appetite, weight loss, decreased production performance, decreased carcass and eggshell quality, liver injury, inhibition of animal immune function and carcinogenesis. Aflatoxin has immunosuppressive properties [25]. Intake of contaminated feed will increase the susceptibility to infection and reduce the immunity of vaccines. AFB1 mainly affects cellular immunity. It can reduce the total number of lymphocytes, especially the total number of circulating activated lymphocytes, inhibit the production of lymphocytes and damage the delayed hypersensitivity and graft-versus-host reaction of skin [26]. AFB1 can also reduce the lysis of natural killer cells and the function of macrophages, such as thiophene swallowing activity, intracellular killing or production of oxidative free radicals [27]. In vitro analysis of mouse peritoneal macrophages exposed to AFB1 showed that the expression of IL-lα and IL-6 or TNF-α increased [28]. Blood lymphocytes of pigs fed with food containing AFB1 feed contaminant were catalyzed by mitogens, and the expression of IL-lP decreased while the expression of IL-10 increased [29]. In addition, studies have shown that AFB1 affects swine growth performance, apparent total tract digestibility and intestinal health, seriously impairing the development of the swine industry [30].

LncRNA plays an important role in many life activities, such as dose compensation effect, epigenetic regulation, cell cycle regulation and cell differentiation regulation [31]. Similarly, microRNA (miRNA) plays a variety of important regulatory roles in cells. Like transcription factors, miRNA regulates gene expression and plays a great role in cell differentiation, biological development and disease occurrence and development, which has attracted more and more attention from researchers [32]. RNA-seq data analysis of mRNA, microRNA and lncRNA provides new clues for gene expression profile and transcriptional regulation in animal cells in response to mycotoxin exposure, and helps to detect biomarkers and drug targets for predicting and controlling mycotoxin contamination [33]. The expressions of lncRNA and miRNA are analyzed by lncRNA microarray, which proves that Zearalenone (Zea) and imprinted lncRNAs are closely related to reproduction and development [34]. Zhang et al. showed that Zearalenone (ZEA) can activate the JAK2–STAT3 signaling pathway through the two lncRNAs MSTRG.22680 and MSTRG.23882 to induce cell apoptosis [35]. ZEN causes toxicological effects by regulating the expression of miRNA and miRNA target genes [36]. Some reports have focused on the role of ncRNAs (miRNA and lncRNA) in AFB1-induced toxicity, especially the relationship between AFB1 and HCC [37]. ADAMTS4, the targeted mir-1268a gene, is affected by pre miRNA polymorphism to reveal the risk of AFB1 related hepatocellular carcinoma(HCC) [38]. In the toxicological study of AFB1, it was also found that AFB1 can affect the expression of miRNAs and lncRNA in the liver, result in liver fat deposition and hepatocyte apoptosis, and induce hepatotoxicity [21]. Our results revealed that AFB1 exposure affected the expression of miRNAs such as ssc-miR-181a, ssc-miR-331-3p and ssc-miR-342 and affected the expression of lncRNAs such as MSTRG.24315 and MSTRG.80767.

AFB1 can affect the expression of many genes. Chemokines are small proteins that control a variety of tissue functions, including cell recruitment and activation under homeostatic and inflammatory conditions. CXCL8 (Interleukin-8) is a member of the chemokine family and acts on CXCR1 and CXCR2 receptors. CXCL8 and its receptors help eliminate pathogens but may also contribute significantly to disease-related processes, including tissue damage, fibrosis, angiogenesis and tumorigenesis [39]. IL-8 is related to a variety of inflammation and chemotaxis and participates in the occurrence of many diseases. The main biological function of IL-8 is to contribute to the chemotaxis of neutrophils, T lymphocytes and basophil (Basophils) during inflammation, and its chemotaxis are different in different cells [40]. GADD45G protein is a stress protein that responds to the environment. As a stress-sensitive factor, it plays an important role in response to toxic and non-toxic stress responses. It also plays an important regulatory role in many cell functions such as DNA repair, cell cycle regulation and senescence, toxic stress response of genes, inducing cell cycle arrest and apoptosis [41,42]. After AFB1 treatment, as one of the major mediators of the inflammatory response, CXCL8 was upregulated with the highest fold change [43]. In addition, AFB1 exposure induced the expression of Cxcl8 and Gadd45g genes in 3D4/2 cells (Figure 5) in our study, leading to cell inflammation, DNA repair, cell cycle arrest and apoptosis, which is consistent with the results of Figure 2F and Figure 3E. Similarly, the mRNA level of Il6 in the liver of broilers exposed to AFB1 was significantly higher than that of the control group [44].

The *Nfkb1* gene is considered to be anti-apoptotic. In liver cells, increased expression of Nfkb1 has been shown to upregulate other inflammatory genes, such as Tnfa and Il6 [45,46]. However, improper activation of NF-κB is associated with a variety of inflammatory diseases, while persistent inhibition of NF-κB leads to improper development of immune cells or delayed cell growth [47]. AFB1 exposure affected the development of macrophages by inhibiting NF-κB (Figure 7). RRAS2 is necessary for the proliferation of human CLL cells. Rras2 encodes a protein that binds to the plasma membrane and plays an important role in activating the signal transduction pathway that controls cell proliferation. RRAS2 is associated with the BCR in leukemic cells and is required for human CLL cell proliferation [48]. The treatment of AFB1 decreased the expression of RRAS2, thus inhibiting the proliferation of cells (Figure 1C and Figure 7). It is worth noting that studies have shown that curcumin successfully alleviated AFB1-induced oxidative stress, inflammation and apoptosis in broiler liver by regulating the expression of lncRNA [49]. This suggests that our study may provide a therapeutic target for the swine industry to control AFB1 toxicity.

## 4. Materials and Methods

### 4.1. In Vitro AFB1 Treatment of Porcine Alveolar Macrophages

AFB1 (AFB1, A832707, Macklin, Shanghai, China) was dissolved in Dimethyl sulfoxide (DMSO) and stored at −20 °C until use. Porcine alveolar macrophages (3D4/2, ATCC: CRL-2845) were cultured in 96-well plates or 6 cm culture dishes (Corning, 430166, New York, NY, USA) for AFB1 treatment at the concentration of 40 μg/mL.

### 4.2. EdU Staining for Proliferation

An EdU Assay/EdU Staining Proliferation Kit (Beyotime, C00755, Shanghai, China) was used to detect and quantify cell proliferation in porcine alveolar macrophage cells using flow cytometry. Proliferating cells were stained for incorporated EdU against total DNA content using Hoechst.

### 4.3. TUNEL Staining

Cells were collected after 24 h of AFB1 treatment and then fixated by 4% Paraformaldehyde. The One Step TUNEL Apoptosis Assay Kit (Beyotime, C1086, Shanghai, China) was used to examine apoptosis cells according to the instructions. After sealing with anti-fluorescence quenching sealing solution, cells were observed under a fluorescence microscope (Olympus, BX51, Tokyo, Japan). TUNEL-positive cell rates were counted and analyzed using IPWIN software (Meedia Cybernetics, Rockville, MD, USA).

### 4.4. RNA Extraction and Sequencing of Whole Transcriptome RNA

The total RNA from cells was extracted using RNAprep Microkit pure (Aidlab, RN07, Shanghai, China) according to the manufacturer’s instructions. The Illumina TruSeq™ RNA preparation kit (Illumina, San Diego, CA, USA) was used to prepare samples, and the Novogene (Beijing, China) HiSeq 4000 platform was used for sequences.

### 4.5. Pipeline of Data Processing

FastQC software (http://www.bioinformatics.bbsrc.ac.uk/projects/fastqc/, accessed on 25 April 2022) and Fastp software (https://github.com/OpenGene/fastp/, accessed on 25 April 2022) were used to analyze the quality control of sequencing data and eliminate the low-quality reads from the raw data. The clean reads of samples were mapped using STAR software for mRNA. SAMtools was also used to remove reads not mapping in a proper mate-pair, and the featureCounts software was used to assign sequence reads to genomic features (Figure S1A).

### 4.6. Screening for Candidate lncRNAs

Preliminary filtering was done based on “class_code” type. We then used Coding Potential Calculator (CPC) [50], Coding–Non-coding Index (CNCI) [51] and PfamScan software (http://xfam.org/, accessed on 25 April 2022) to identify lncRNAs [52] (Figure S1B).

### 4.7. Discovering Differentially Expressed Genes (DEGs) and RNA Target Prediction

Differentially expressed lncRNAs (DElncRNAs) and miRNAs (DEmiRNAs) were assessed using the R Bioconductor/DESeq2 package (https://support.bioconductor.org/, accessed on 25 April 2022). The targeting relationship of DElncRNAs and DEmRNAs (Differentially expressed mRNA) was predicted using the R package Hmisc (https://github.com/harrelfe/Hmisc, accessed on 25 April 2022). We then used FEELnc (v 0.1.1) (https://github.com/tderrien/FEELnc, accessed on 25 April 2022) to find mRNAs cis-regulated by lncRNAs [53]. The target genes of the miRNAs were predicted using TargetScan, miRanda and RNAhybrid [54,55].

### 4.8. GO Classification and KEGG Enrichment Analysis

Gene Ontology (GO) enrichment analysis was performed using the “org.Ss.eg.db” database (http://bioconductor.org/packages/release/data/annotation/html/org.Ss.eg.db.html, accessed on 25 April 2022) to convert the gene SYMOL to ENTREZID. The Kyoto Encyclopedia of Genes and Genomes (KEGG) pathways was analyzed using the R Bioconductor/Pathview package (http://bioconductor.org/developers/how-to/buildingPackagesForBioc/, accessed on 25 April 2022).

### 4.9. RT-qPCR

The whole transcriptome RNA from cells was extracted with the EASYspin Plus cellular RNA rapid extraction kit (Aidlab, RN2802, Beijing, China), and reverse transcription was conducted using the MiRcute Plus miRNA First-Strand cDNA Kit (Tiangen, KR211-01, Beijing, China). The MiRcute Plus miRNA qPCR Kit (SYBR Green) (Tiangen, FP411-01, Beijing, China) was employed (primers are shown in Table S1). Relative quantitative PCR data analysis was performed using the difference multiple = 2^−ΔΔct^ method.

### 4.10. Immunofluorescence Staining

Cells were treated with AFB1 for 24 h in order to perform immunofluorescence staining. The following primary antibodies were used: CXCL8 (IL8, DF6998, Affinity Biosciences, Cincinnati, OH, USA), GADD45G (GADD45G, DF2376, Affinity Biosciences, Cincinnati, OH, USA) and NFKB1 (NFKB1, BF0466, Affinity Biosciences, Cincinnati, OH, USA), RRAS2 (RRAS2, DF9840, Affinity Biosciences, Cincinnati, OH, USA). Goat anti-Rabbit IgG (H + I) (Beyotime, A0521, Nantong, China) was used as the second antibody. The methods of immunofluorescence staining followed the published methods [56].

### 4.11. Statistical Method

The differences between mean values were statistically tested using Student’s t test or one-way ANOVA followed by the Tukey test for multiple comparisons. Comparisons were considered significant at *p* < 0.05 (*) and *p* < 0.01 (**).

## Data Availability

Not applicable.

## Supplementary Materials

The following supporting information can be downloaded at: https://www.mdpi.com/article/10.3390/toxins14060373/s1, Figure S1: Data preprocessing and Candidate lncRNA identification; Figure S2: Target genes of DEmRNAs, DEmiRNAs, and miRNA-mRNA network.; Table S1: The sequences of primers.
