# Supplementary material for: Whole-Transcriptome Analysis of Non-Coding RNA Alteration in Porcine Alveolar Macrophage Exposed to Aflatoxin B1
Source: Toxins (Basel). 2022 May 27;14(6):373. doi: 10.3390/toxins14060373 (PMC9230535; doi:10.3390/toxins14060373)
Supplement: Supplementary file 1 [file toxins-14-00373-s001.zip › toxins-1722220-supplementary.pdf]

toxins

Supplementary Materials

Whole-transcriptome analysis of non-coding RNA alteration in porcine alveolar macrophage exposed to Aflatoxin B1

Huhe Chao, Haohai Ma, Jiadong Sun, Shuai Yuan, Peiyu Dong, Aihong Zhao, Lan Li, Wei Shen and Xifeng Zhang

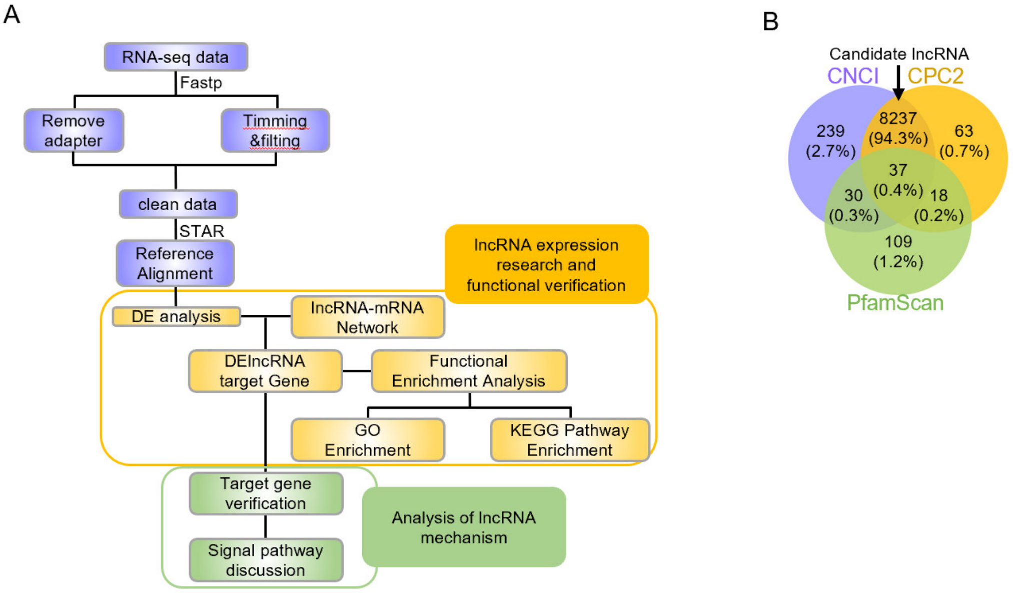

Figure S1. (A) Data preprocessing pipeline for mRNA and lncRNA. **(B)** Candidate lncRNA identification with coding potential assessment of CNCI, CPC2, and PfamScan; the black arrow refers to the final candidate lncRNA.

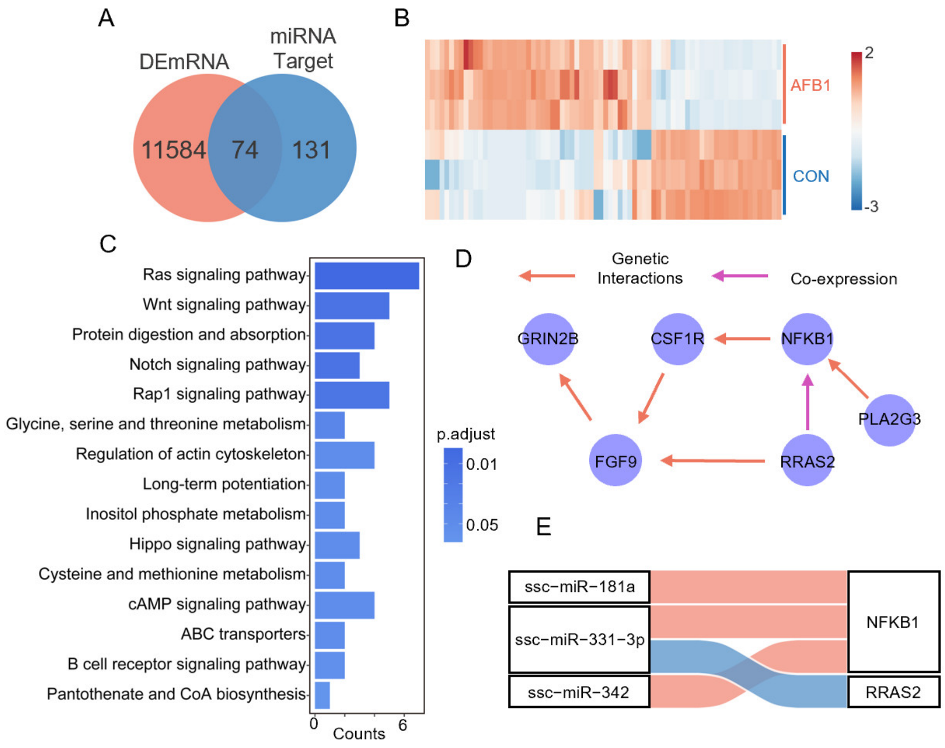

Figure S2. (A) The Venn diagram demonstrating the relationship between DEmRNAs and target genes. **(B)** The heatmap displaying the expression levels of DEmiRNAs target genes. **(C)** GO enrichment analysis of DEmiRNAs target genes. **(D)** Genetic interactions and co-expression networks of key genes in the Ras signaling pathway. **(E)** Sankey diagram of the miRNA-mRNA network.

Table S1. The sequences of primers.

Primers	Sequences
ssc-miR-181a	AACATTCAACGCTGTCGGTGAGTT
ssc-miR-331-3p	GCCCCTGGGCCTATCCTAGAA
ssc-miR-342	TCTCACACAGAAATCGCACCCGTCA
U6-R	TGGAACGCTTCACGAATTTGCG
U6-F	GGAACGATACAGAGAAGATTAGC
